# Increased expression of complement C3c, iC3b, and cells containing CD11b or CD14 in experimentally induced psoriatic lesion

**DOI:** 10.1093/cei/uxae009

**Published:** 2024-02-04

**Authors:** Dina Rahkola, Rauno J Harvima, Ilkka T Harvima

**Affiliations:** Department of Dermatology, University of Eastern Finland and Kuopio University Hospital, Kuopio, Finland; Department of Dermatology, University of Eastern Finland and Kuopio University Hospital, Kuopio, Finland; Department of Dermatology, University of Eastern Finland and Kuopio University Hospital, Kuopio, Finland

**Keywords:** psoriasis, isomorphic reaction, complement C3, CD11b, CD14

## Abstract

Psoriasis is a chronic inflammatory skin disease with a characteristic isomorphic reaction, i.e. the Köbner reaction, induced by slight epidermal trauma. In this study, the tape-stripping technique was used to induce the development of Köbner reaction in 18 subjects with psoriasis. Eight subjects developed a positive reaction. To study the early cellular changes, skin biopsies were taken at the baseline and subsequent time points of 2 h, 1 d, 3 d, and 7 d for the immunostaining of complement C3c, iC3b, and cells expressing complement receptor 3 (CD11b/CD18; a receptor of iC3b) or CD14. The results show that the positive Köbner reaction is associated with rapid (2 h–1 d) and sustained (3–7 d) increase in the expression of epidermal C3c and iC3b and dermal C3c. In addition, there was a positive correlation between CD11b^+^ and CD14^+^ cells in baseline and 2 h–1 d biopsies with a subsequent increase in CD11b^+^ and CD14^+^ cells in 3–7 d biopsies in the Köbner-positive group. In the Köbner-negative group, only a transient increase in epidermal iC3b at 2 h–1 d, as well as rapid (2 h–1 d) and sustained increase (3–7 d) in dermal iC3b and CD14^+^ cells, was observed. In experiments with cultured monolayer keratinocytes, a slight cell damage already at 30 mJ/cm^2^ ultraviolet B irradiation led to increased expression of C3c, but not iC3b. Therefore, there are marked differences between Köbner groups in respect to the expression of C3c, iC3b, and cells expressing CD11b or CD14. Of note is the rapid and sustained increase in epidermal C3c and iC3b in the positive Köbner reaction.

## Teaser

The isomorphic psoriatic reaction is especially associated with rapid and sustained increase in the expression of epidermal C3c and iC3b and dermal C3c. In addition, the significant correlation between CD11b^+^ and CD14^+^ cells in 0 d and 2 h–1 d biopsies, as well as the subsequent increase in CD11b^+^ and CD14^+^ cells in 3–7 d biopsies, in the Köbner-positive group suggests that there are differences between Köbner groups in respect to dermal cellular environment already before the induction of the isomorphic psoriatic reaction. The expression of C3c immunoreactivity in cryosections from a representative Köbner-positive patient with psoriasis on day 0 (a), day 1 (b), and day 7 (c).The immunohistochemical staining of CD11b^+^ cells in cryosections from a Köbner-positive patient with psoriasis on day 0 (d) and day 7 (e).

## Graphical Abstract

**Graphical Abstract:**
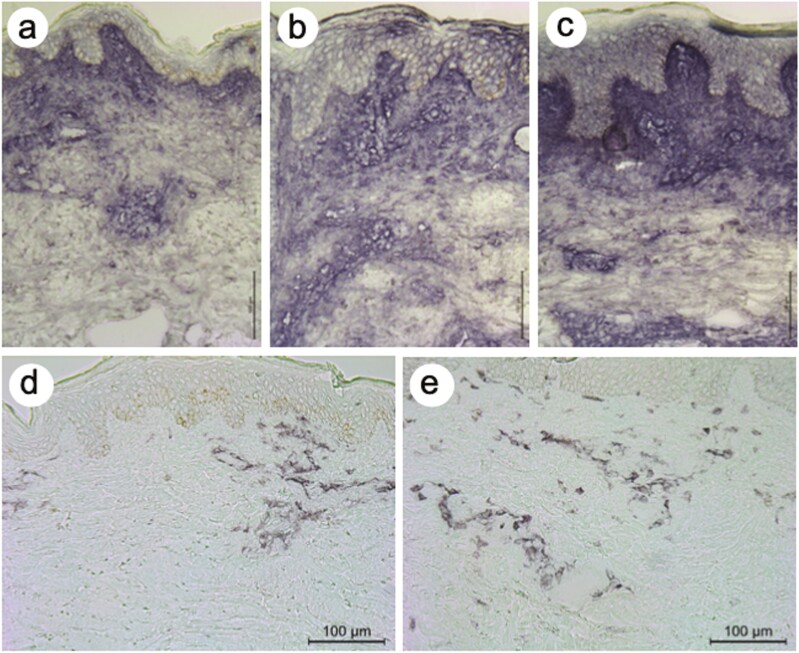

## Introduction

Chronic inflammation and epidermal hyperplasia are typical features of psoriatic pathogenesis that is characterized by the accumulation of T helper 1 (Th1) and 17 (Th17) cells, antigen-presenting cells, neutrophils, and mast cells [1, 2]. The isomorphic psoriatic reaction, i.e. the Köbner reaction, is another typical feature in psoriasis that develops on the nonlesional psoriatic skin as a response to slight trauma 7–14 d later. The rupture of epidermis is reportedly an essential factor for the Köbner reaction, but dermal involvement is also needed [3]. In our previous studies, the positive Köbner reaction induced by the tape-stripping technique was associated with a rapid and transient decrease in epidermal interleukin (IL)-33 immunostaining [4], higher percentages of dermal IL-6^+^ mast cells, and appearance of dermal cells immune positive for IL-6 receptor and IL-33 [5]. In contrast, the negative Köbner reaction was associated with the dermal appearance of cells expressing the Forkhead Box P3 (FoxP3) and their increased morphological contacts with tryptase^+^ mast cells [6].

Complement factor C3 is the essential factor of the complement system that is involved in the innate immunity. Upon activation of the complement system, C3 is converted to anaphylatoxin C3a and C3b that is further converted to iC3b. The inactivated product iC3b represents, in fact, an immunologically active molecule that is covalently attached to C3b-acceptor sites, e.g. on cell membranes, and it is the ligand of the complement receptor CR3 (CD11b/CD18, Mac-1) [7]. In addition to plasma as a well-known source, C3 is produced locally by cutaneous resident cells as well, including keratinocytes and mast cells [8–10]. The CD11b receptor is expressed on neutrophils, monocytes, and myeloid-derived suppressor cells (MDSCs). Therefore, it can have a proinflammatory role in disease pathogenesis, though it may have immunosuppressive functions in some disease conditions, such as cancer [11–13]. Increased numbers of CD11b^+^ cells have been detected in the psoriatic lesion [14, 15]. The expression of CD11b on granulocytes and monocytes has been reported to correlate with pustule formation in psoriasis [16]. Therefore, CD11b associates with psoriasis though it is poorly investigated in psoriasis compared to other integrin receptors, such as CD11a.

Previously, we have found that the percentage of mast cells expressing C3c immunoreactivity is increased in the lesional skin of patients with psoriasis or basal cell carcinoma, and mast cells were suggested to be a marked source for C3 degradation products, including iC3b. Furthermore, the percentage of C3c^+^ mast cells correlated to the number of CD11b^+^ cells [17]. There are no previous studies that investigate the C3-iC3b-CD11b axis in the early phases of psoriasis. Therefore, we utilized again the unique isomorphic psoriatic reaction induced by tape-stripping for investigating the very early molecular and cellular changes in the C3-iC3b-CD11b axis [4–6]. For this, skin biopsies were collected at the time points of 0 h, 2 h, 1 d, 3 d, and 7 d. Cryosections were prepared to investigate the appearance of the immunoreactivity of C3c, iC3b neoantigen, and CD11b. In addition, CD14 was analysed as it is a pattern-recognition receptor expressed by monocytes and macrophages and can work in collaboration with CD11b [11, 18]. The results show differences between patients with positive Köbner reaction and those with negative one.

## Materials and methods

### Induction of the psoriatic lesion and collection of skin biopsies

The patients with chronic plaque psoriasis have been described previously [4–6]. The patients comprised 18 subjects (4 females and 14 males, aged 24–77 years) with the psoriasis area and severity index between 1.8 and 18.6. After obtaining the consent, the Köbner reaction was induced using the tape-stripping technique [4–6]. Briefly, an area (about 2.5 cm × 5–7 cm) on the lateral aspect of the arm was tape-stripped using an adhesive tape for about 30–40 times until the skin revealed slight redness. The first (day 0) 4-mm punch biopsy was taken from a skin site that was just outside of the tape-stripped area. The study subjects were randomly divided into two groups: in the first group, the 4-mm skin biopsies were taken at the time points of 2 h and 3 d and in the second group at 1 d and 7 d. The subjects were evaluated in the follow-up visit about 2–2.5 weeks later. The subject was judged to belong to the Köbner-positive group (8 subjects out of 18) if an identifiable reddish change could be seen on the tape-stripped area, but outside of the skin biopsy sites. The patients had not received any effective systemic or local treatment during the preceding month. After removal, the skin biopsies were immediately embedded in optimal cutting temperature (OCT) compound (Miles Scientific, Naperville, IL, USA) and frozen for preparing 5-µm-thick cryosections as described [4–6]. The methods used were approved by the Ethics Committee of Kuopio University Hospital, Kuopio, Finland, as described in our previous studies [4–6].

### Immunohistochemical staining of CD11b, CD14, C3c, and iC3b

For the staining of CD11b (CD126), CD14, iC3b, or C3c, skin cryosections were fixed in cold acetone followed by immunohistochemistry using 0.5 µg/ml anti-human CD11b mouse monoclonal antibody (mAb; clone C11b/660, Novus Biologicals, Abingdon, UK), 0.3 µg/ml anti-CD14 mouse mAb (clone TÜK4, Dako Denmark A/S, Glostrup, Denmark), 0.06 µg/ml anti-human iC3b (neo) mouse mAb (catalog number A209, IgG2bκ, Quidel, San Diego, CA, USA), or 0.75 µg/ml anti-human C3c rabbit polyclonal antibody (pAb; catalog number A 0062, Dako) and Vectastain Elite ABC kit [17, 19]. An unrelated mouse or rabbit immunoglobulin G (IgG) was used as the control. The number of CD11b^+^ or CD14^+^ cells was counted on 3 cryosections per slide with a microscope in a dermal area beneath the epidermis using a 40× objective and ocular grid [17, 19]. The results are expressed as cells/mm^2^. After the initial assessment, the staining intensity of iC3b in the epidermis and the upper dermis was analysed using a semiquantitative scoring. In the upper dermis, score 1 denotes weak staining, score 2 denotes moderate staining, and score 3 denotes strong staining. In the epidermis, the following scoring was used: score 0 denotes no staining or remarkably weak staining, score 0.5 denotes weak staining, score 1 denotes moderate staining that is either darker or wider than the weak staining, score 2 denotes strong focal staining, and score 3 denotes strong staining in almost the whole epidermal length. The same semiquantitative scorings were used in the analysis of C3c. Three cryosections per biopsy were photographed using a 20× objective.

### Sequential double-staining method for iC3b and tryptase

The technique to clarify whether iC3b is related to tryptase^+^ mast cells has previously been described [17]. Briefly, the photographed cryosections stained with 0.06 µg/ml anti-iC3b mAb and Vectastain Elite ABC kit (with biotinylated horse anti-mouse IgG) were restained immunohistochemically using 0.36 µg/ml rabbit polyclonal anti-tryptase antibody [20] and Vectastain ABC-AP KIT (alkaline phosphatase, rabbit IgG, with biotinylated goat anti-rabbit IgG). The stainings were controlled using mouse or rabbit IgG. The sections were then rephotographed exactly at the same sites using a 20× objective.

### Immunocytochemical staining of C3c and iC3b of cultured keratinocytes irradiated with ultraviolet B

Keratinocytes were isolated from healthy human foreskins of infant donors, and the cells were cultured in Keratinocyte-SFM™ serum-free medium (Life Technologies, Paisley, UK), supplemented with 5 ng/ml epidermal growth factor, 50 μg/ml bovine pituitary extract, 100 U/ml penicillin, and 100 μg/ml streptomycin [21]. The cells were seeded in wells of 4-well chamber slides using this complete medium, and after reaching about 70% confluence, the cells were washed twice with Dulbecco’s phosphate-buffered saline (PBS) followed by incubation in incomplete SFM-medium (without epidermal growth factor and bovine pituitary extract) at 5% CO_2_ and 37°C for 1–2 h. Then, the cells were irradiated once with 0, 30, 60, 90, and 120 mJ/cm^2^ ultraviolet B (UVB) using DuaLight™ UVA/UVB Phototherapy device (TheraLight™, Inc., Carlsbad, CA, USA), as described [22]. After a 24-h incubation, the cells were washed with PBS and fixed in cold acetone for 10 min. The first set of glass slides was stained immunocytochemically using 0.75 µg/ml rabbit polyclonal anti-C3c pAb and the second one using 0.06 µg/ml mouse monoclonal anti-iC3b using Vectastain ABC-AP KIT or Vectastain Elite ABC KIT for visualization. All culture sites on the glass slides were photographed at least at 10 randomly chosen fields using a 20× objective to determine the percentage of C3c^+^ keratinocytes. Only cells with clearly dark C3c immunostaining were counted. The experiment was performed twice.

### Statistics

The results were analysed using two-tailed paired *t*-test or mixed model analysis, and *P* < 0.05 was considered statistically significant. A correlation between variables was tested using the Spearman correlation test.

## Results

### The score of C3c in the epidermis and dermis

**Figure 1.**
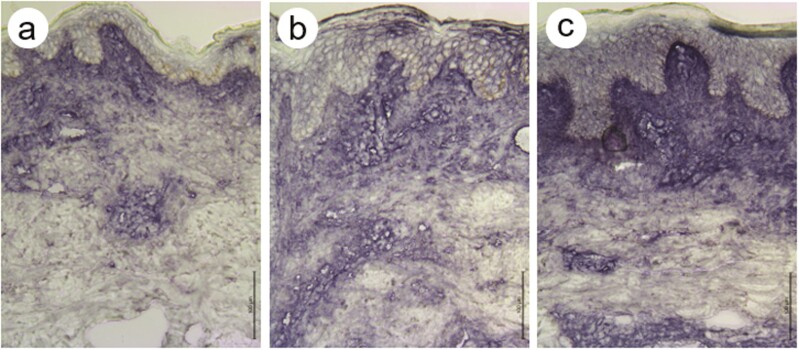
The expression of C3c immunoreactivity in cryosections from a representative Köbner-positive patient with psoriasis on day 0 (**a**), day 1 (**b**), and day 7 (**c**). The cryosections were stained immunohistochemically using rabbit anti-human C3c pAb. Note the increase in epidermal C3c immunostaining in days 1 and 7 compared to day 0. Also, note the increase in the immunostaining of C3c in the upper and papillary dermis from day 0 (a) to day 1 (b) and 7 (c). The micrographs were taken using a 20× objective (scale bar = 100 μm).

The score of C3c was significantly increased both in the epidermis and upper dermis in 2 h–1 d biopsies, and further so in the epidermis in 3–7 d biopsies, in the Köbner-positive group (Table 1; Fig. 1). In contrast, no such increase was observed in the Köbner-negative group.

**Table 1.** The score of C3c immunostaining in the epidermis and dermis in the Köbner-positive and Köbner-negative groups of psoriatic patients

		C3c score at different time points		
		0 d	2 h–1 d	3–7 d
Köbner-positive	Epidermis	0.75 ± 0.38	1.50 ± 0.53*	1.88 ± 0.99**
	Dermis	1.63 ± 0.52	2.38 ± 0.74*	2.25 ± 0.46
		(*n* = 8)	(*n* = 8)	(*n* = 8)
Köbner-negative	Epidermis	1.05 ± 0.37	1.45 ± 1.11	1.67 ± 1.20
	Dermis	2.20 ± 0.42	2.00 ± 0.82	1.89 ± 0.78
		(*n* = 10)	(*n* = 10)	(*n* = 9)

The results are expressed as the mean ± SD. *P < 0.05 and **P < 0.01 (mixed model analysis) when comparing the score of C3c in 2 h–1 d or in 3–7 d biopsies to that in corresponding 0 d biopsies.

### The score of iC3b immunostaining in the dermis

**Figure 2.**
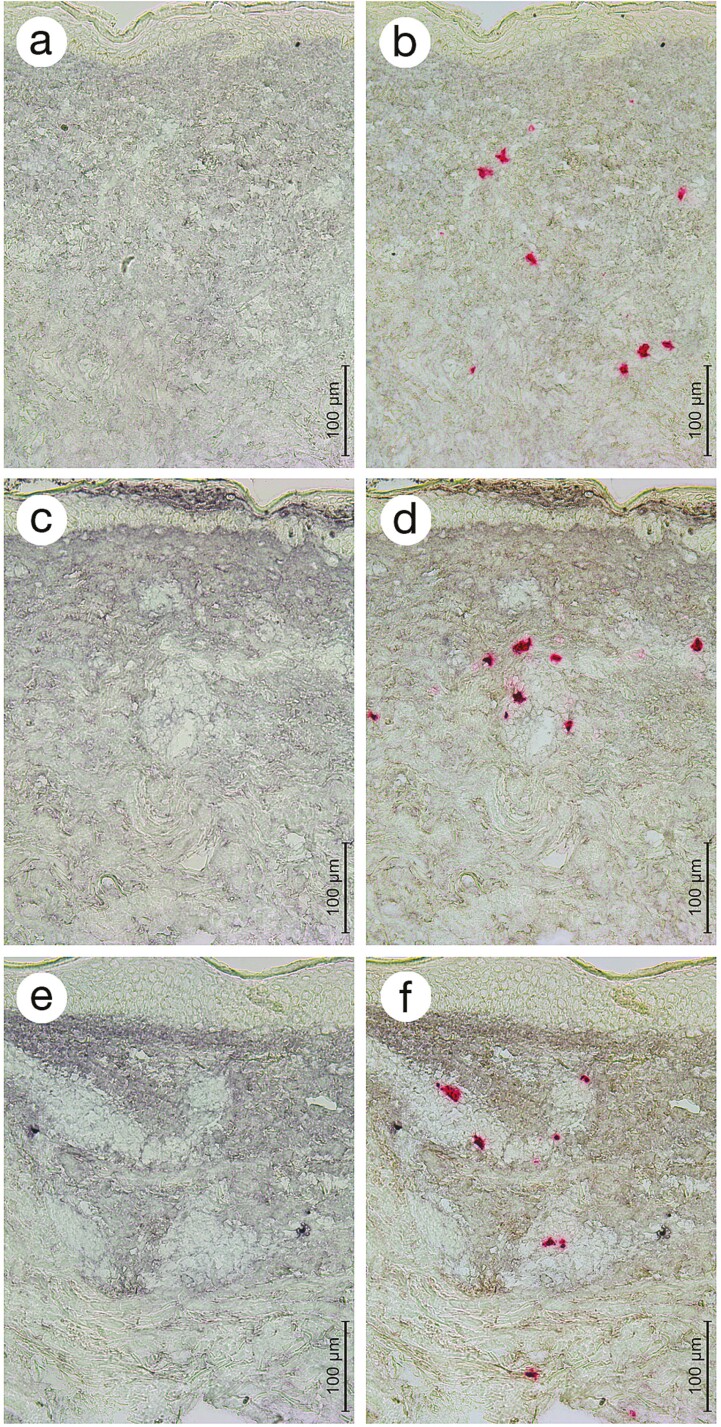
Sequential double-staining method for iC3b and tryptase in cryosections from a representative Köbner-negative patient with psoriasis on day 0 (**a, b**), day 1 (**c, d**), and day 7 (**e, f**). First, the cryosections were stained immunohistochemically for iC3b (black staining product; a, c, e). After photographing, the same cryosections were stained immunohistochemically for mast cell tryptase (red staining product; b, d, f). Note the increase in dermal iC3b immunostaining on day 1 (c) and 7 (e). Note also the focal iC3b staining in the upper epidermis (c, d). The dermal iC3b staining is not markedly associated with tryptase^+^ mast cells (b, d, f). The micrographs were taken using a 20× objective (scale bar = 100 μm).

The score of iC3b was significantly higher in both 2 h–1 d and 3–7 d biopsies compared to that in corresponding 0 d biopsies in the Köbner-positive group (Table 2). In the Köbner-negative group (Table 2; Fig. 2), the score of iC3b was also significantly increased in 2 h–1 d and in 3–7 d biopsies compared to corresponding 0 d biopsies. No significant differences between Köbner-positive and Köbner-negative groups were observed.

**Table 2.** The score of iC3b immunostaining in the epidermis and dermis in the Köbner-positive and Köbner-negative groups of psoriatic patients

		iC3b score at different time points		
		0 d	2 h–1 d	3–7 d
Köbner-positive	Epidermis	0.3 ± 0.3	1.1 ± 0.6*	0.9 ± 0.6**
	Dermis	1.4 ± 0.5	2.4 ± 0.7^¶^	2.1 ± 0.6^¶¶^
		(*n* = 8)	(*n* = 8)	(*n* = 8)
Köbner-negative	Epidermis	0.2 ± 0.3	1.5 ± 1.0***	0.7 ± 0.4
	Dermis	1.4 ± 0.5	2.4 ± 0.8^¶^	2.0 ± 1.0^¶¶^
		(*n* = 10)	(*n* = 10)	(*n* = 9)

The results are expressed as the mean ± SD. *P = 0.004, **P = 0.038, and ***P < 0.001 (mixed model analysis) when comparing the epidermal iC3b scores in 2 h–1 d or in 3–7 d biopsies to those in corresponding 0 d biopsies. ^¶^*P* < 0.0005 and ^¶¶^*P* < 0.01 when comparing the dermal iC3b scores in 2 h–1 d or in 3–7 d biopsies to those in corresponding 0 d biopsies.

After restaining of cryosections for mast cell tryptase (Fig. 2), it was noted that the cytoplasm of mast cells is not markedly immunostained for iC3b. In addition, the dermal iC3b immunoreactivity did not show any clear association with mast cells or their close extracellular environment suggesting that the iC3b detected is mostly derived from other cells than mast cells.

### The score of iC3b immunostaining in the epidermis

In the Köbner-positive group, the epidermal iC3b score was significantly higher in 2 h–1 d and in 3–7 d biopsies than in 0 d biopsies (Table 2). In contrast, in the Köbner-negative group, the epidermal iC3b score was significantly higher in 2 h–1 d biopsies, but not so in 3–7 d biopsies, compared to 0 d biopsies. However, no significant differences were observed between Köbner-positive and Köbner-negative groups.

An interesting finding was the focal iC3b immunostaining in the upper epidermis in 1 d biopsies in both the Köbner-negative (*n* = 2) and Köbner-positive (*n* = 2) groups (Fig. 2). However, this iC3b accumulation was transient as it was not detected in 7 d biopsies in either Köbner groups and only in one 3 d biopsy in the Köbner-positive group.

### CD11b^+^ cells in the upper dermis

**Figure 3.**
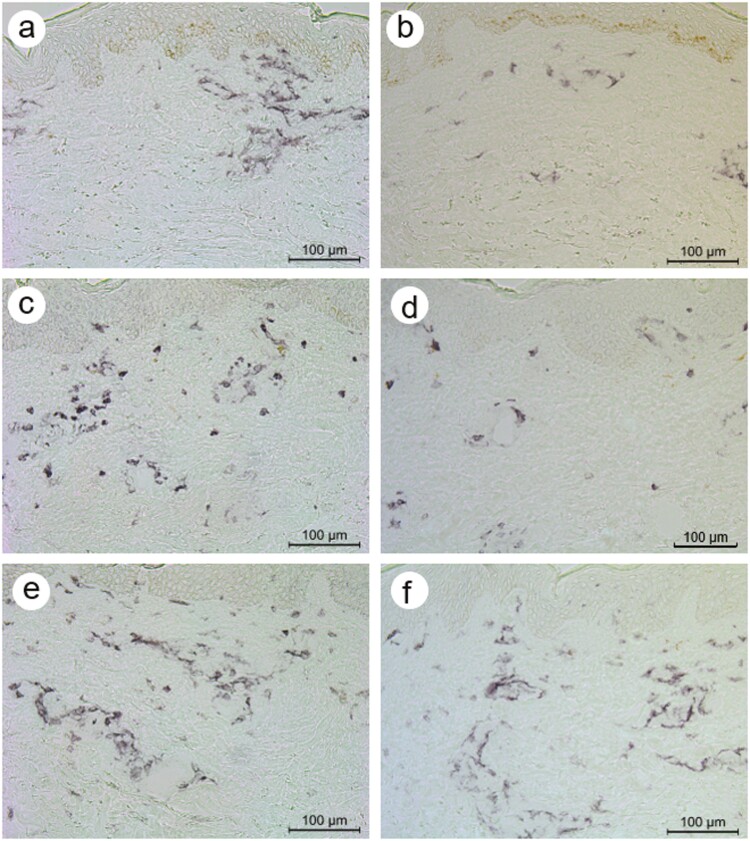
Immunohistochemical staining of CD11b-positive and CD14-positive cells in cryosections from a Köbner-positive patient with psoriasis on day 0 (**a, b**), day 1 (**c, d**), and day 7 (**e, f**). The cryosections (a, c, e) were stained for CD11b and the cryosections (b, d, f) for CD14. The micrographs were taken using a 20× objective (scale bar = 100 μm).

In the Köbner-positive group, the number of CD11b^+^ cells was significantly increased in 3–7 d biopsies compared to that in corresponding 0 d biopsies (Table 3; Fig. 3). In the Köbner-negative group, statistically no significant changes in CD11b^+^ cell numbers were observed, though there was a trend towards increased cell numbers (Table 3).

**Table 3.** The number of cells showing CD11b or CD14 immunoreactivity in the upper dermis in the Köbner-positive and Köbner-negative groups of psoriatic patients

		Immunopositive cells at different time points (cells/mm^2^)		
		0 d	2 h–1 d	3–7 d
Köbner-positive	CD11b	245 ± 117	386 ± 182	421 ± 130*
	CD14	298 ± 202	315 ± 152	551 ± 125**
		(*n* = 8)	(*n* = 8)	(*n* = 8)
Köbner-negative	CD11b	354 ± 148	433 ± 126	550 ± 245
	CD14	296 ± 103	398 ± 78^¶^	473 ± 174^¶¶^
		(*n* = 10)	(*n* = 10)	(*n* = 9)

The results are expressed as the mean ± SD. *P = 0.016 and **P = 0.040 (paired *t*-test) when comparing the cell numbers in 3–7 d biopsies to those in corresponding 0 d biopsies. ^¶^*P* = 0.006 and ^¶¶^*P* = 0.031 when comparing the cell numbers in 2 h–1 d or in 3–7 d biopsies, respectively, to those in corresponding 0 d biopsies.

### CD14^+^ cells in the upper dermis

The number of CD14^+^ cells was increased in number in the upper dermis in both Köbner groups (Table 3; Fig. 3). However, there are differences between groups: CD14^+^ cells were significantly increased already in 2 h–1 d biopsies in the Köbner-negative group, whereas the increase was significant only in 3–7 d biopsies in the Köbner-positive group (Fig. 3).

### The correlation between the number of CD11b^+^ cells and CD14^+^ cells

In the Köbner-positive group, the correlation between CD11b^+^ and CD14^+^ cells was significant in 0 d (*r* = 0.789, *P* = 0.020) and 2 h–1 d biopsies (*r* = 0.796, *P* = 0.018), but not so in 3–7 d biopsies (*r* = −0.473, *P* = 0.236). In contrast, there were no such significant correlations in the Köbner-negative group.

### The correlation between dermal iC3b score and CD11b^+^ cells

In the Köbner-positive group, there was a correlation (*r* = 0.695) with borderline significance (*P* = 0.056) between the score of iC3b and CD11b^+^ cells in 0 d biopsies, but not so in 2 h–1 d and 3–7 d biopsies. In the Köbner-negative group, no correlations were found between these parameters in any biopsy group.

### The correlation between dermal C3c score and CD11b^+^ cells

The dermal score of C3c immunoreactivity did not show any statistical association with CD11b^+^ cells in the Köbner-positive or Köbner-negative group.

### The correlation between C3c and iC3b in the dermis and epidermis

In the Köbner-positive group, the dermal score of C3c correlated significantly to the dermal score of iC3b (*r* = 0.742, *P* = 0.035) in 2 h–1 d biopsies, but not in 0 d or 3–7 d biopsy groups. In the Köbner-negative group, no such significant correlations were observed in any biopsy groups. No correlations were seen between these parameters in the epidermis.

### The expression of C3c and iC3b immunoreactivity in keratinocytes irradiated with UVB

**Figure 4.**
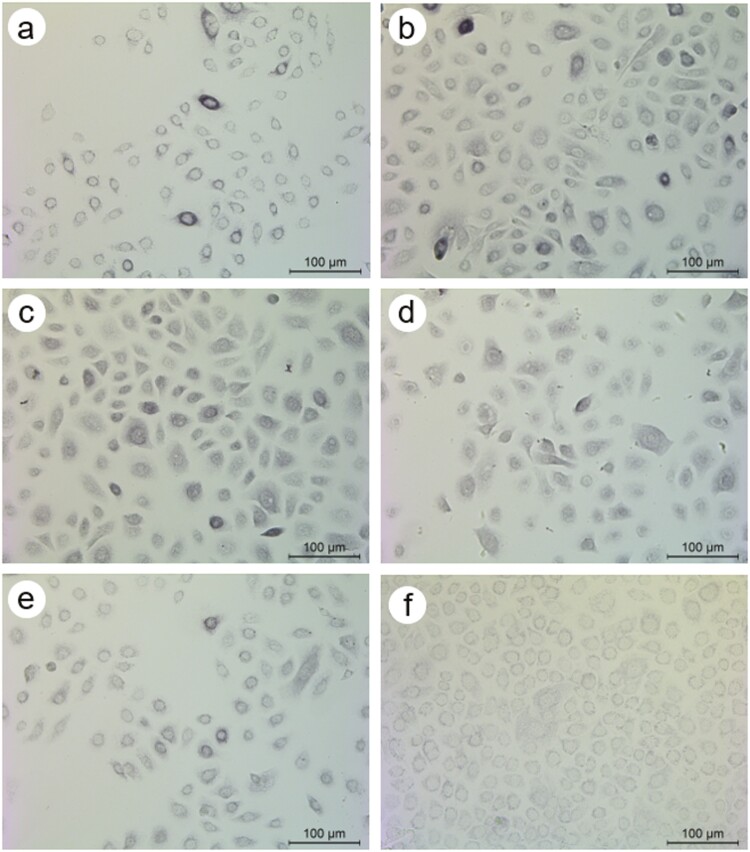
The expression of C3c immunoreactivity in keratinocytes irradiated with UVB. Control cells were cultured without irradiation (**a**). The other cells were irradiated using 30 mJ/cm^2^ UVB (**b**), 60 mJ/cm^2^ UVB (**c**), 90 mJ/cm^2^ UVB (**d**), or 120 mJ/cm^2^ UVB (**e**). After the irradiation and consequent 24-hour incubation, the cells were stained immunocytochemically for C3c (a–e). In addition, control non-irradiated cells were immunostained using an IgG control (**f**). Note the decrease in the staining intensity of C3c in cells irradiated with 60–120 mJ/cm^2^ UVB (c–e). The micrographs were taken using a 20× objective (scale bar = 100 μm).

Cultured monolayer keratinocytes were damaged by using different doses of UVB irradiation. The cell number of keratinocytes increased significantly after 30 mJ/cm^2^ UVB, but at higher UVB doses, it was switched to a decrease suggesting cell damage (Table 4). The expression of C3c immunoreactivity in keratinocytes followed a similar trend, that is, the percentage of C3c^+^ cells increased after 30 mJ/cm^2^ UVB, but it declined thereafter (Table 4; Fig. 4). Of note, there was no detectable iC3b staining in keratinocytes—not even after irradiation.

**Table 4.** The number of keratinocytes and the percentage of C3c^+^ keratinocytes irradiated with UVB

	Keratinocytes irradiated with UVB (mJ/cm^2^)				
	0	30	60	90	120
Cell number	162 ± 91	207 ± 43*	142 ± 51**	122 ± 36*	117 ± 49*
C3c (%)	0.41 ± 0.79	1.74 ± 2.05*	1.68 ± 2.48*	2.40 ± 3.51*	0.51 ± 1.14^¶^
	(*n* = 109)	(*n* = 111)	(*n* = 116)	(*n* = 100)	(*n* = 106)

The results are expressed as the mean ± SD. *P < 0.001, and **P = 0.012 (linear mixed model analysis) when comparing the cell number in a micrograph or the percentage of C3c^+^ keratinocytes irradiated with 30, 60, 90, or 120 mJ/cm^2^ UVB to control cells without irradiation (0 mJ/cm^2^ UVB). ^¶^*P* < 0.001 when comparing the percentage of C3c^+^ keratinocytes irradiated with 120 mJ/cm^2^ UVB to those irradiated with 30, 60, or 90 mJ/cm^2^ UVB. ‘*n*’ denotes the number of micrographs taken using a 20× objective.

## Discussion

By using this same biopsy material, a transient reduction in epidermal IL-33 expression has been observed at 2 h, 1 d, and 3 d in the Köbner-positive, but not Köbner-negative group [4], suggesting that this ‘alarmin’ is released rapidly resulting in consequent activation of inflammatory cells [5, 23, 24].

Complement C3 has been found to be constitutively produced by human keratinocytes, and its expression is increased by proinflammatory cytokines, such as IL-1α, interferon-γ, and tumor necrosis factor-α [10]. The expression of C3c immunoreactivity in cultured monolayer keratinocytes was verified in this study, too, and the slight cell damage with 30 mJ/cm^2^ UVB even increased their C3c expression. In this study, both the epidermal and dermal expression of C3c increased significantly in 2 h–1 d biopsies in the Köbner-positive, but not Köbner-negative group, a finding which suggests a proinflammatory role for C3, supported by findings in a mouse model [25]. This strong increase in the epidermis and upper dermis suggests that C3c immunoreactivity is derived from, e.g. keratinocytes, mast cells and blood microcirculation. C3 is known to be a subject of proteolytic processing resulting in the formation of C3a and C3b [7]. The anaphylatoxin C3a may act as an autocrine stimulus by enhancing C3 synthesis in keratinocytes through its receptor [complement C3a receptor (C3aR)] [26], and it has been detected even in the scales of psoriasis [27]. In addition, C3a can induce IL-8 secretion from endothelial cells [28] and polymorphonuclear leukocytes [29], and IL-8 is a known attractant of neutrophils [30].

C3b is covalently bound to C3b-acceptor sites and it is cleaved further to iC3b by complement factor I in cooperation with co-factors [7]. Small amounts of functional complement factor I have previously been shown to be constitutively produced by cultured human keratinocytes and the production is increased by interferon-γ [31]. However, no iC3b expression was detected in cultured keratinocytes in this study, not even after UVB irradiation, which may be due to low expression of complement factor I and/or the absence of co-factors. Nevertheless, a rapid increase in iC3b expression was found in 2 h–1 d biopsies in the epidermis and dermis in the Köbner-positive group, and the expression level was sustained in 3–7 d biopsies. Similarly, the expression of iC3b increased in the epidermis and dermis in the Köbner-negative group, but it started to disappear in the epidermis in 3–7 d biopsies. Therefore, the positive Köbner reaction was associated with rapid and sustained upregulation in the expression of both C3c and iC3b in the epidermis. In the negative Köbner reaction, the sustained upregulation of iC3b in the dermis without apparent increase in dermal C3c implies that there is enhanced processing of C3 to iC3b that in turn can act as a tolerance-inducing mediator through binding to CD11b on antigen-presenting cells [32]. The strong focal increase in iC3b in the upper epidermis in both Köbner groups in 1 d biopsies is interesting, and it may be related to inherent epidermal response to superficial trauma and opsonization of, e.g. necrotic keratinocytes [33] and/or to microbes. Also, it may be related to the characteristic neutrophil accumulation in the upper epidermis of psoriasis [34].

The number of mast cells is higher in the lesional than nonlesional psoriatic skin, but the chymotryptic serine proteinase chymase is partially inactivated presumably by its protease inhibitors [35]. Because chymase can degrade complement C3 and C3a [9], the partial inactivation of chymase in the developing psoriatic lesion [35, 36] is supposed to leave the C3 protein and its fragments sufficiently active.

In the Köbner-positive group, but not significantly so in the Köbner-negative group, the number of dermal CD11b^+^ cells was significantly increased in 3–7 d biopsies. In addition, there was a significant positive correlation between CD11b^+^ and CD14^+^ cells in 0 d and 2 h–1 d biopsies suggesting at least some co-localization of CD11b and CD14 in the same cells. Further, there was a correlation with borderline significance (*P* = 0.056) between the score of iC3b and CD11b^+^ cells in 0 d biopsies in the Köbner-positive group. Therefore, the dermal cellular environment between Köbner groups can be different already in the very early lesion, even before the induction of the isomorphic lesion. The possible role of iC3b in the recruitment of CD11b^+^, CD14^+^ cells of the monocytic lineage is supported by previous findings that the deposition of iC3b at the dermal–epidermal junction after UVB irradiation is followed by CD11b^+^ cell accumulation to the same site [37]. In addition, in a transwell assay *in vitro*, iC3b stimulated migration of monocytic cells from peripheral blood mononuclear cells [19]. However, the interaction of iC3b with CD11b can lead to immunosuppression or induction of tolerance [32, 37–39], and/or the accumulation of CD11b^+^CD14^+^HLA-DR^−^/^low^CD15^−^ myeloid-derived suppressor cells of the monocytic lineage (M-MDSC), and the distinction between MDSCs and proinflammatory CD11b^+^ neutrophils or CD11b^+^, CD14^+^ monocytes by immunohistochemistry is difficult without functional assays of isolated cells [12, 13]. Therefore, it is possible that the dermal C3c-iC3b-CD11b/CD14 axis in the Köbner-positive group may not necessarily represent a proinflammatory process, but just a failed immunosuppressive attempt to control inflammation. This hypothesis is supported by the result that the expression of dermal iC3b and, as well as at least that of CD14 (and a trend in CD11b), increased already in 2 h–1 d biopsies in the Köbner-negative group, and the increase was sustained in 3–7 d biopsies.

In conclusion, the present results suggest that the isomorphic psoriatic reaction is especially associated with a rapid and sustained increase in the epidermal expression of C3c and iC3b as well as with the increase in dermal expression of C3c. In addition, the significant correlation between CD11b^+^ and CD14^+^ cells in 0 d and 2 h–1 d biopsies, as well as the subsequent increase in CD11b^+^ and CD14^+^ cells in 3–7 d biopsies, in the Köbner-positive group suggests that there are differences between Köbner groups in respect to dermal cellular environment already before the induction of the isomorphic psoriatic reaction.

## Data Availability

The data can be available on a reasonable request through the corresponding author.

## Abbreviations

C3aR: complement C3a receptor
CD: cluster of differentiation
CR: complement receptor
FoxP3: forkhead box P3
IgG: immunoglobulin G
IL-33: interleukin 33
mAb: monoclonal antibody
MDSC: myeloid-derived suppressor cell
M-MDSC: myeloid-derived suppressor cell of the monocytic lineage
OCT: optimal cutting temperature
pAb: polyclonal antibody
PBS: phosphate-buffered saline
Th: T helper
UVB: ultraviolet B.
